# Efficacy prediction of bacteriophage-antibiotic combinations against *Staphylococcus aureus* biofilms using planktonic bacteria

**DOI:** 10.3389/fmicb.2025.1706083

**Published:** 2026-01-02

**Authors:** Mariëlle Verheul, Elizabeth Schonkeren-Ravensbergen, Eva M. Bus, Mark G. J. de Boer, Rob G. H. H. Nelissen, Bart G. Pijls, Peter H. Nibbering

**Affiliations:** 1 Leiden University Center for Infectious Diseases (LUCID), Laboratory of Infectious Diseases, Leiden University Medical Center (LUMC), Leiden, Netherlands; 2 Department of Orthopedics, Leiden University Medical Center (LUMC), Leiden, Netherlands

**Keywords:** phage ISP, antibiotic, mature biofilm, repeated exposure, implant-related infections, receiver operating characteristic (ROC) curve, predictive models, translational microbiology

## Abstract

**Introduction:**

Phage-antibiotic combinations (PAC) may be effective in eradicating clinical, mostly biofilm-associated, infections. As the efficacy of PAC largely depends on the phage and antibiotic used, such combinations should be screened before their clinical application. Unfortunately, testing the efficacy of PAC on mature biofilms is laborious. This study aimed to assess whether the effects of PAC on biofilm-encased bacteria can be predicted by testing their effects on planktonic counterparts.

**Methods:**

Methicillin-sensitive *Staphylococcus aureus* (MSSA, clinical isolate) in mid-logarithmic phase was exposed to (sub)optimal doses of phage ISP combined with antibiotics targeting transcription, protein translation, the cell wall, and the cell membrane. These experiments were followed by studies assessing the effects of up to three daily exposures to PAC on MSSA within seven-day mature biofilms on metal implant mimics. An additional 2-log reduction or an increase in bacterial counts with PAC compared to the most effective single agent (phage or antibiotic) was considered as synergy or antagonism, respectively. Receiver operating characteristic (ROC) curves were used to calculate whether the effects of PAC on planktonic bacteria were comparable to the effects on biofilm-encased bacteria.

**Results:**

The results for planktonic bacteria showed antagonism between ISP and rifampicin or gentamicin, synergism between ISP and ciprofloxacin, clindamycin, or flucloxacillin, and lack of interaction between ISP and daptomycin. Similarly, ISP combined with rifampicin or gentamicin displayed antagonism on biofilm-encased MSSA, whereas ISP and ciprofloxacin, clindamycin, flucloxacillin, or high-dose daptomycin induced synergy. Notably, two to three consecutive daily exposures to PAC were necessary to reduce biofilm-encased MSSA maximally. Testing PAC on planktonic bacteria predicted antagonistic effects on biofilms (area under the curve (AUC) = 0.95), but did not predict synergistic effects (AUC = 0.30) after 3 days of exposure to the respective phage-antibiotic concentrations.

**Discussion:**

Together, PAC testing on planktonic bacteria provides a valuable first-line screening tool for clinicians treating biofilm-associated infections.

## 1 Introduction

Biofilm infections on metal implants, often caused by *Staphylococcus aureus* (*S. aureus*) (Zeller et al., 2018; Knebel et al., 2020), not only hugely impact the patient’s quality of life but also pose a tremendous burden on healthcare systems due to their association with antibiotic treatment failure (Fernandes and Dias, 2013). Prolonged antibiotic treatment combined with surgical debridement, or even extensive implant revision surgeries, is often insufficient to eliminate these infections. At the same time, the infection itself as well as these often complex treatment regimens and sequelae contribute to the patient’s psychological distress, pain, and disability, while also resulting in high healthcare costs (Knebel et al., 2020; Scheper et al., 2021; Morgenstern et al., 2021; Woffenden et al., 2023).

One of the major causes of failure of antibiotic treatment and surgical debridement is bacterial biofilm formation on the metal implant surface. Biofilm-encased bacteria are protected from the effects of host immune cells and antibiotics, as the biofilm matrix hinders their access, penetration, and efficacy (Høiby et al., 2010; Bjarnsholt et al., 2011). Within the biofilm, gradients of nutrients, oxygen, and pH result in heterogeneous bacterial subpopulations with different, often reduced, antibiotic sensitivities (Høiby et al., 2010).

The tolerance of these biofilm-associated infections to antibiotic treatment underlines the importance of exploring alternative treatment modalities, such as bacteriophage (phage) therapy. Lytic phages are viruses that specifically infect and kill bacterial strain(s) within a species by attaching to the receptor of the bacterial cell, inserting their genome into the cytoplasm, and taking over the bacterial replication and translation machinery to produce new phages (Kutter, 2004). The lytic enzymes of phages cause bacterial lysis, leading to bacterial cell death and the release of the phage progeny.

Although primarily used in Eastern Europe, lytic phages have been utilized to treat bacterial infections since the early 1900s (Sulakvelidze et al., 2001). Currently, phage therapy is still considered experimental in Western Europe and is only permitted for compassionate use, i.e., for patients with life-threatening infections when no other therapies are available (Hitchcock et al., 2023). Due to their relative specificity, phages have to be selected for the bacterial strain causing the infection. Many studies have reported positive treatment outcomes with phage therapy for difficult-to-treat infections, including bone and joint infections (Clarke et al., 2020).

Further, personalized phage therapy combined with standard-of-care antibiotics was more effective than antibiotic treatment alone in achieving positive treatment outcomes (Pirnay et al., 2024). One of the most frequently used phages in this cohort was phage ISP, which has a relatively broad activity against a range of *S. aureus* strains (Vandersteegen et al., 2011). Previously, ISP as a single agent was ineffective at eliminating *S. aureus* within seven-day mature biofilms on a metal implant mimic (Verheul et al., 2024). It remains elusive whether ISP encodes exopolysaccharide depolymerases that can disintegrate the biofilm matrix (Vandersteegen, 2013). While phage-antibiotic synergy has been observed *in vitro* with other phages against biofilm-encased *S. aureus* (Tkhilaishvili et al., 2018; Kunz Coyne et al., 2024), the effect of ISP combined with antibiotics has remained unexplored for such biofilms on metal implant surfaces.

The synergistic potential for phage-antibiotic therapy depends on the antibiotic and phage being used (Gu Liu et al., 2020). For instance, antibiotics that target the bacterial cell wall or membrane by compromising the cell wall stability can enhance bacterial lysis with a lower number of phages compared to controls, explaining potential synergy between these antibiotics and phages. In contrast, antibiotics that inhibit protein synthesis may diminish the production of phage proteins by the bacterial machinery, which may lead to reductions in phage assembly, replication, and subsequent lysis of the bacteria by the phage (Abedon, 2019; Kunz Coyne et al., 2024). Correctly pairing phages and antibiotics to enhance bacterial eradication is a crucial step before their introduction into the clinic. Currently, this pairing involves testing phage-antibiotic combinations (PAC) on planktonic phase bacteria or on biofilm models (Kunz Coyne et al., 2024). Assessing these interactions using planktonic phase bacteria is more practical, since testing PAC on clinically relevant biofilm models (cultured for at least 5 days (Cazander et al., 2010)) is particularly laborious. However, it remains unclear how the effects of PAC on planktonic phase bacteria relate to the effects on clinically relevant biofilm models.

Therefore, this study aimed to assess whether the effects of PAC on biofilm-encased bacteria can be accurately predicted by testing their effects on planktonic counterparts. For this purpose, ISP was used in combination with clinically relevant antibiotics targeting bacterial transcription, protein synthesis, the cell wall, and the cell membrane of *S. aureus*.

## 2 Materials and methods

### 2.1 Bacteria and bacterial culture

Methicillin-sensitive *S. aureus* (MSSA; LUH15393, clinical isolate obtained from a patient with prosthetic joint infection (PJI), sensitive for all antibiotics used in this study) were frozen in 20% glycerol and stored at −70 °C until use, i.e., spreading bacterial stock on trypticase soy agar plates with 5% sheep blood (43009, Biomerieux) and overnight incubation at 37 °C. Mid-logarithmic phase bacteria were obtained by taking single colonies from this plate and subsequent incubation in tryptone soy broth (TSB, CM0129, Oxoid Ltd) for 2.5 h (200 rpm, 37 °C). The bacteria were centrifuged (1,000 x*g*, 10 min) and washed with phosphate-buffered saline (PBS, pH 7.4). Based on the OD_600nm_, bacteria were diluted to 10^6^ CFU/mL (for killing assays of planktonic bacteria) or 10^7^ CFU/mL (for seven-day biofilm culture) in brain-heart infusion (BHI, CM1135, Oxoid Ltd) broth.

### 2.2 Antibiotics

Phage-antibiotic interactions largely depend on the antibiotic’s mechanism of inhibition (Gu Liu et al., 2020). Hence, this study selected antibiotics targeting different bacterial processes or structures to combine with phage ISP, i.e., transcription (rifampicin, ciprofloxacin), protein synthesis (gentamicin, clindamycin), the cell wall (flucloxacillin), or the cell membrane (daptomycin). Rifampicin (4 mg/mL in DMSO, 13292-46-1), ciprofloxacin (5.12 mg/mL in Milli-Q water, 86393-32-0), daptomycin (1 mg/mL in DMSO, 103060-53-3), and flucloxacillin (10 mg/mL in Milli-Q water, 1847-24-1) were stored at −20 °C, gentamicin (50 mg/mL, 1405-41-0) at 4 °C (all from Sigma Aldrich), and clindamycin (12 mg/mL, RVG 123224, Added Pharma) at room temperature until use. Antibiotics were pre-diluted in Milli-Q water and thereafter diluted in BHI. The BHI of experiments using daptomycin was supplemented with 50 mg/L Ca^2+^ following EUCAST guidelines (Turnidge et al., 2020). These antibiotics were selected based on the susceptibility profile of the MSSA strain. The minimal bactericidal concentration (MBC; the concentration that eliminates 99.9% of the inoculum (ESCMID, 1998)) of the antibiotics amounted to 15.6 μg/L (rifampicin), 0.8 μg/mL (ciprofloxacin), 4 μg/mL (gentamicin), 8 μg/mL (clindamycin), 0.25 μg/mL (flucloxacillin), and 2 μg/mL (daptomycin) for our MSSA strain. Antibiotic concentrations used in the planktonic killing assay amounted to 1.95–15.6 μg/L (rifampicin), 0.2–0.8 μg/mL (ciprofloxacin), 0.5–4 μg/mL (gentamicin), 1 and 4 μg/mL (clindamycin), 0.06–0.125 μg/mL (flucloxacillin), and 0.5–2 μg/mL (daptomycin). Antibiotic concentrations used in the biofilm assay amounted to 3.9 and 7.8 μg/L (rifampicin), 0.4 and 0.8 μg/mL (ciprofloxacin), 4, 8, and 16 μg/mL (gentamicin), 1 and 4 μg/mL (clindamycin), 0.125 and 1 μg/mL (flucloxacillin), and 6, 30, and 60 μg/mL (daptomycin). These concentrations were based on the sensitivity of planktonic MSSA and active peak plasma concentrations that can be reached in humans, i.e., approximately 8.8 μg/mL [rifampicin, dosage of 600 mg IV (Gianni, 1983; Fage et al., 2023)], 3.1 μg/mL [ciprofloxacin, dosage of 400 mg IV (Lettieri et al., 1992; Abdulla et al., 2020)], 16.2 μg/mL [gentamicin, dosage of 5.9 mg/kg IV (Perrier et al., 2025; Bailey and Briggs, 2004)], 4.0 μg/mL [clindamycin, dosage of 600 mg IV (Zeller et al., 2010; Dorn et al., 2020; Bouazza et al., 2012)], 1.1 μg/mL [flucloxacillin, dosage of 1,000 mg oral (Gardiner et al., 2018)], and 93.9 μg/mL [daptomycin, dosage of 6 mg/kg IV (Garonzik et al., 2016; Rice and Mendez-Vigo, 2009)].

### 2.3 Bacteriophage (phage) ISP

Fresh stock of phage ISP (Herelleviridae family, *Kayvirus* genus, morphotype myovirus; dsDNA, non-segmented), effective against MSSA (LUH15393) as confirmed previously (Verheul et al., 2024), was propagated in methicillin-resistant *S. aureus* [MRSA; LUH14616, sequence type 247, NCCB 100829; kindly provided by Dr. S. Croes (Department of Medical Microbiology, Maastricht University Medical Center, Maastricht, the Netherlands)] using the double agar overlay method (Verheul et al., 2024; Adams, 1959). ISP at a stock concentration of 10^10^ plaque-forming units (PFU)/mL was stored at 4 °C until use. The phage titer in the stock solution and test samples was determined by mixing 1 mL of 10-fold phage dilutions in PBS with 100 μL of mid-logarithmic MSSA (10^9^ CFU/mL in PBS) and 3.5 mL of semi-solid Luria Bertani (LB; LB broth (244620) with 6% LB agar (244520), Difco™ BD Biosciences) at 45 °C. Then, this mixture was poured onto LB agar plates and incubated overnight at 37 °C to enumerate the PFU/mL.

### 2.4 Killing of planktonic bacteria

MSSA (10^6^ CFU/mL) in BHI were incubated with (sub)optimal concentrations of antibiotics, ISP, and combinations thereof in a v-bottom polypropylene 96-wells plate (655201, Greiner Bio-One) for 24 h (37 °C, 90 rpm) after sealing the plate with a non-breathable plastic sealing film (WB 2–3830, Westburg). Suboptimal concentrations were used to identify enhanced bacterial elimination with PACs compared to the single agents, while optimal phage concentrations were used to identify antagonism. Phage titers of 10^2^ PFU/mL (multiplicity of infection (MOI) = 0.0001) and 10^6^ PFU/mL (MOI = 1) were defined as suboptimal and optimal, respectively. After exposure, bacteria were centrifuged (1,000 x*g*, 10 min), and 25 μL of the supernatant was collected to determine the phage titer as mentioned before. The bacterial pellet was resuspended, and 10 mM ammonium iron (II) sulfate hexahydrate (FAS, 100 mM, v/v, F1543, Sigma Aldrich) was added to neutralize residual phage activity. FAS did not affect bacterial counts. Bacterial suspensions were serially diluted in 0.9% saline, plated on Mueller-Hinton (MH) agar (CM0337, Oxoid Ltd) plates, and incubated overnight at 37 °C to determine the bacterial load microbiologically. Medium controls were included to check for possible contamination, and each experiment was repeated three times in triplicate.

### 2.5 Biofilm formation and exposure

Mature biofilms were obtained by applying 100 μL of MSSA at 10^7^ CFU/mL in BHI onto the metal implant mimic (medical grade titanium-6% aluminum-7% niobium [Ti-6Al-7Nb; TAN iso5832/11 disks, diameter 5 mm, height 1.5 mm, handled as described previously (Verheul et al., 2021); kind gift from Dr. T. F. Moriarty (AO Research Institute, Davos, Switzerland)]) in a flat-bottom polystyrene 96-wells plate, sealing plates with a breathable rayon sealing film (391–1262, VWR), and incubation for 7 days at 37 °C (static, without medium refreshment) in a humidified environment. Medium controls were included to check for possible contamination. Subsequently, biofilms were exposed daily to antibiotics, ISP (10^8^ PFU/mL, MOI = 1), or their combination in BHI for one, two, or three consecutive days. These exposures (100 μL per biofilm) were refreshed every 24 h, and the effects of the exposures were assessed after each day. BHI was used as a medium control, and the plates were sealed with non-breathable plastic sealing film. Before and after each exposure, biofilms were washed twice with 0.9% saline to remove planktonic bacteria. At each time point, biofilms were harvested by sonicating the disks (40 kHz, 10 min) in 100 μL 0.9% saline with 10 mM FAS (v/v). Bacterial suspensions were serially diluted in 0.9% saline and plated on MH agar to enumerate bacterial counts. Each experiment was repeated three times in triplicate.

### 2.6 Data analysis

Bacterial survival data were log-transformed to approximate a normal distribution and to calculate the mean log CFU/mL with 95% confidence intervals using GraphPad Prism 10.2.3. Following international recommendations, 95% confidence intervals were reported rather than *p*-values (Ranstam, 2012; Amrhein et al., 2019). The antibacterial effects of PAC, i.e., reductions in mean CFU/mL compared to the most effective single agent, were considered antagonistic, indifferent, additive, and synergistic if the mean difference was <0-log, ≥ 0 < 1-log, ≥ 1 < 2-log CFU/mL, and ≥ 2-log CFU/mL, respectively (Doern, 2014; Lenhard et al., 2016).

**Figure 1:**
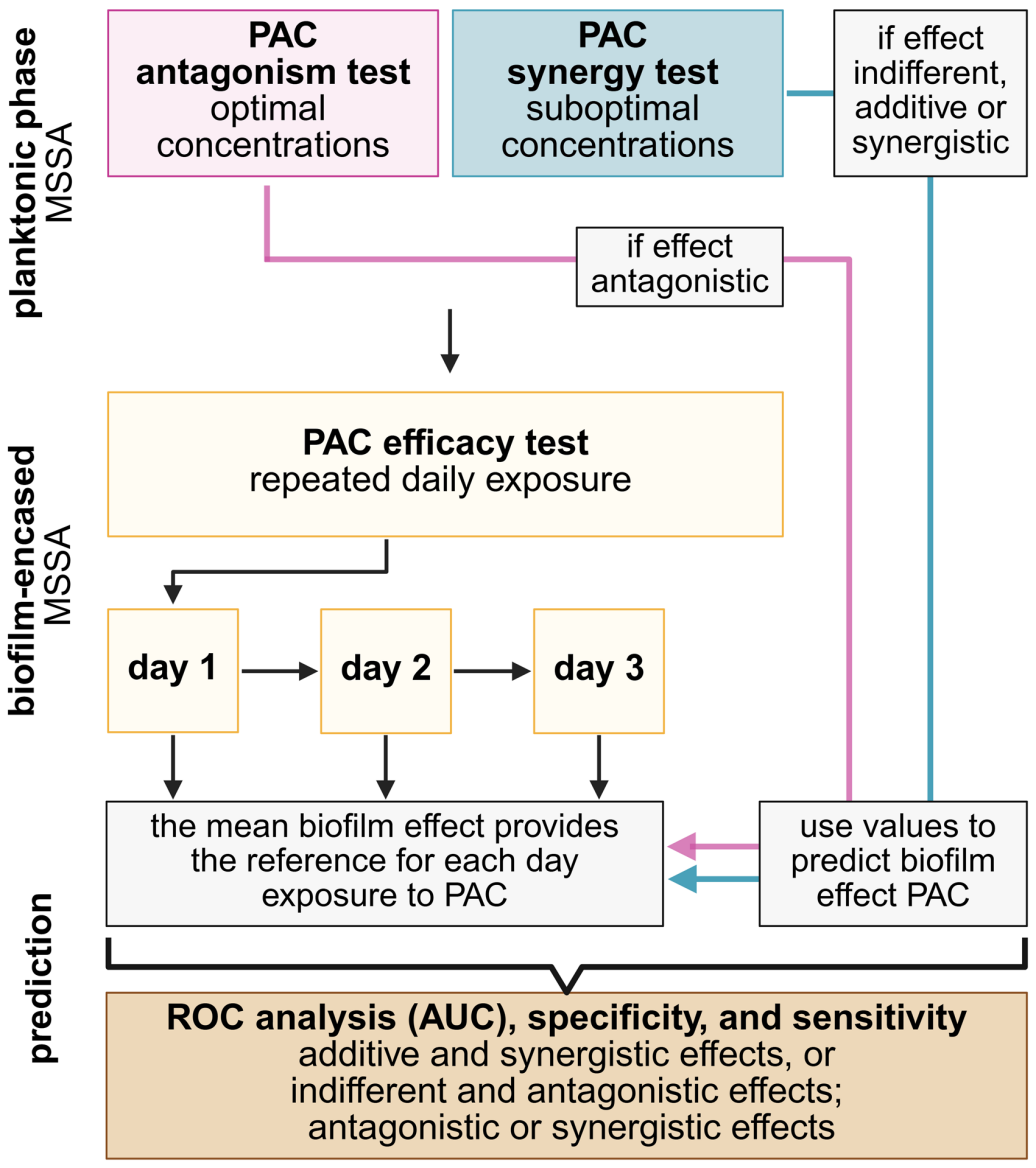
Overview of the experimental set-up to predict the efficacy of phage-antibiotic combinations (PAC) on biofilm-encased methicillin-sensitive *Staphylococcus aureus* (MSSA) using planktonic phase MSSA. Additive and synergistic effects are combined to indicate favorable effects and indifferent and antagonistic effects are categorized to indicate unhelpful effects. Planktonic and biofilm PAC tests are based on microbiological enumeration of bacterial counts (colony forming units (CFU)/mL) upon the exposure to PAC compared to exposure to the single agents. In addition, the planktonic antagonism test evaluated the phage titers (plaque forming units (PFU)/mL) after bacterial exposure to PAC compared to exposure of the phage alone. Figure created using Biorender.com.

Additive and synergistic values were categorized together to indicate favorable effects, while antagonistic and indifferent effects were combined to indicate unhelpful effects of PAC. If PAC was antagonistic to planktonic bacteria, the data points for PAC with optimal ISP concentrations (10^6^ PFU/mL) were utilized to predict the anti-biofilm effect (Figure 1). If additive or synergistic effects of PAC on planktonic bacteria were observed, the values from exposure to suboptimal concentrations of antibiotics combined with ISP (10^2^ PFU/mL) were used to predict the anti-biofilm effect. If the effect of PAC on planktonic bacteria was indifferent, values of 0 were used (this approach was applied for daptomycin). The mean reduction of biofilm-encased bacterial counts for each PAC served as the standard for each exposure day. These values were compared to the predicted values for planktonic bacteria by computing the receiver operating characteristic (ROC) curve, the area under the curve (AUC), and the sensitivity and specificity, using IBM SPSS Statistics (version 30.0.0).

ROC curves, frequently used to determine the accuracy of diagnostic tests, plot the true positive rate (sensitivity) against the false positive rate (1 – specificity) at various thresholds (Altman and Bland, 1994). A straight diagonal line from the lower left to the upper right corner on a ROC curve indicates that the predictions of the model are not better than chance. The AUC reflects the test’s overall performance along the ROC curve. AUC values ≥0.7 and <0.8 were considered acceptable, ≥0.8 and <0.9 excellent, and ≥0.9 outstanding (Hosmer and Lemeshow, 2000). The results of this study were reported according to the STARD guidelines for reporting diagnostic accuracy (Cohen et al., 2016).

## 3 Results

### 3.1 Effect of combinations of phage ISP with transcription-targeting antibiotics on MSSA

#### 3.1.1 Planktonic phase MSSA

**Figure 2:**
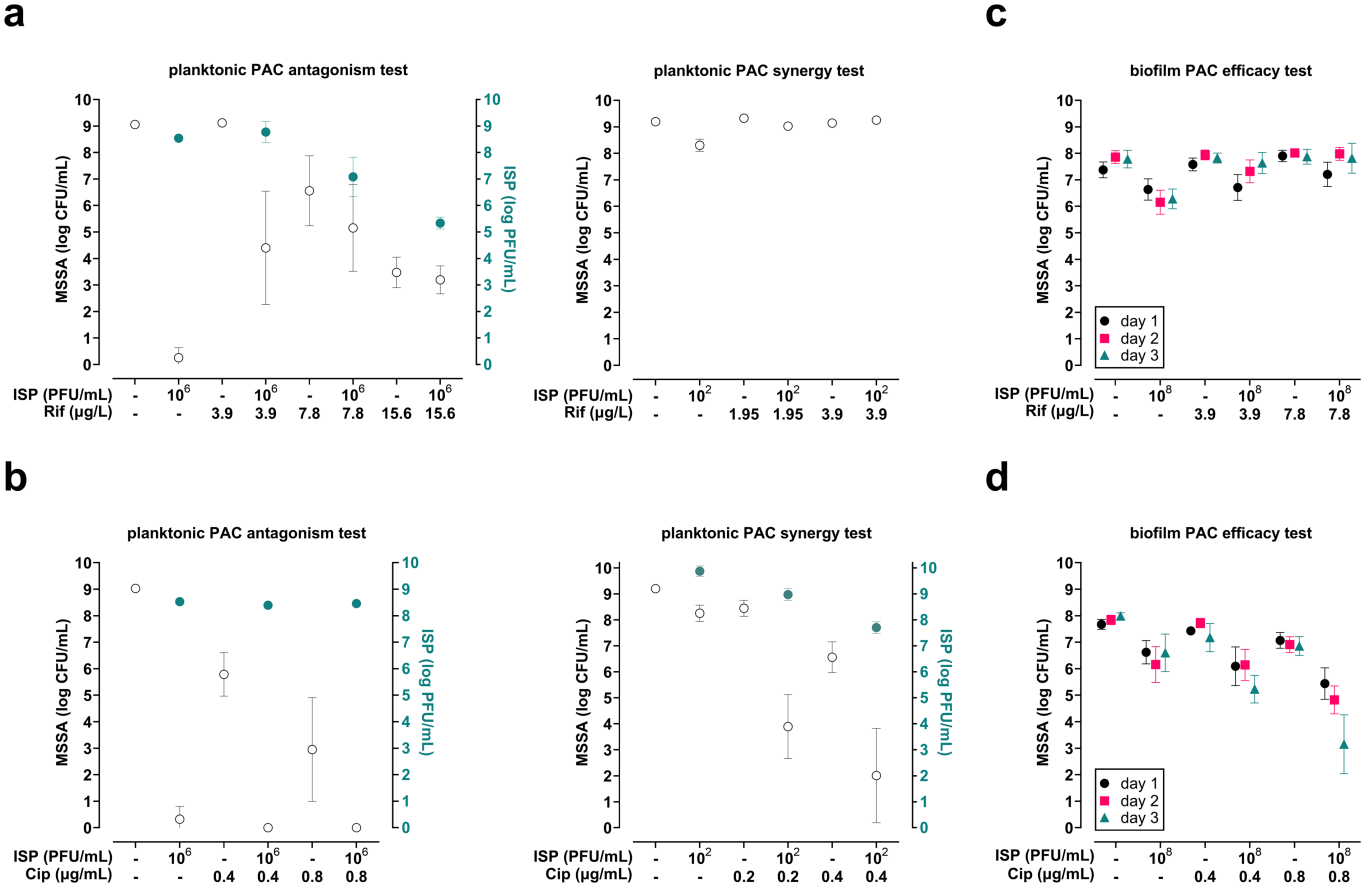
Efficacy of bacteriophage (phage) ISP, antibiotics targeting bacterial transcription, and combinations thereof on planktonic and biofilm-encased methicillin-sensitive *Staphylococcus aureus* (MSSA). The efficacy of phage-antibiotic combinations (PAC) was tested by exposing MSSA in mid-logarithmic phase at 10^6^ CFU/mL to PAC with phage ISP at 10^6^ PFU/mL (multiplicity of infection (MOI) = 1) or ISP at 10^2^ PFU/mL (MOI = 0.0001) in combination with **(a)** rifampicin (Rif) or **(b)** ciprofloxacin (Cip) at suboptimal concentrations. After 24 h exposure (90 rpm, 37 °C), the bacteria were spun down by centrifugation (1,000 *xg*, 10 min). Subsequently, the phage titer (PFU/mL) in the supernatant was determined using the double agar overlay method and the bacterial load in the pellet was assessed microbiologically after resuspension in 0.9% saline supplemented with 10 mM ammonium sulfate (II) hexahydrate (FAS; to neutralize residual phage activity). The open (white) symbols in **a** and **b** indicate the mean CFU/mL and the closed (blue) symbols indicate the mean PFU/mL. In addition, the efficacy of PAC was assessed on MSSA within seven-day mature biofilms on titanium-6% aluminum-7% niobium (Ti-6Al-7Nb) implant mimics. Briefly, biofilms were exposed daily to ISP at 10^8^ PFU/mL and its combination with **(c)** rifampicin or **(d)** ciprofloxacin for up to three consecutive days. Before and after each exposure, planktonic bacteria were removed by two washes with 0.9% saline. Finally, bacteria were harvested from biofilms by sonication (40 kHz, 10 min) in 0.9% saline with 10 mM FAS for microbiological quantification of bacterial counts. Brain-heart infusion broth was used as diluent for all exposures. Results are from at least three independent experiments, each in triplicate. Data were log-transformed and error bars indicate the 95% confidence intervals.

The effects of ISP combined with the transcription-targeting antibiotics rifampicin or ciprofloxacin were first investigated on MSSA in planktonic phase (10^6^ CFU/mL). Exposure of planktonic phase MSSA to ISP (10^6^ PFU/mL, MOI = 1) effectively eliminated MSSA and resulted in increased phage titers. The phage’s antibacterial efficacy was hampered by rifampicin in a dose-dependent manner, which was accompanied by reduced phage titers compared with exposure to ISP alone (Figure 2a). Exposure of MSSA to suboptimal doses of ISP (10^2^ PFU/mL, MOI = 0.0001) and rifampicin did not reduce bacterial counts (Figure 2a). In contrast, ISP (10^6^ PFU/mL, MOI = 1) combined with ciprofloxacin resulted in complete bacterial eradication and phage titers similar to those obtained by ISP alone (Figure 2b), indicating no effect of this antibiotic on the phage’s ability to eliminate MSSA. Ciprofloxacin at 0.2 μg/mL and 0.4 μg/mL combined with ISP (10^2^ PFU/mL, MOI = 0.0001) synergistically reduced bacterial counts in planktonic phase by a mean of 4.4-log (95% CI −5.4 to −3.3) and 4.6-log (95% CI −6.2 to −2.9) CFU/mL, respectively. Of note, the viral titer after exposure to ISP at 10^2^ PFU/mL combined with suboptimal concentrations of ciprofloxacin was lower than that after exposure to ISP alone (Figure 2b). In summary, rifampicin antagonized ISP, while ciprofloxacin was synergistic with ISP in eliminating MSSA in planktonic phase.

#### 3.1.2 Biofilm-encased MSSA

As the efficacy of single exposure to ISP and antibiotics was limited against *S. aureus* in dual-species biofilms (Wang et al., 2024), we determined the effects of daily exposure to ISP (10^8^ PFU/mL) combined with rifampicin or ciprofloxacin for up to three consecutive days on biofilm-encased MSSA. The results revealed that combinations of rifampicin and ISP were completely ineffective against biofilm-encased MSSA (Figure 2c). However, ISP combined with ciprofloxacin at 0.8 μg/mL further reduced biofilm-encased bacterial counts by a mean of 1.2-log (95% CI −1.9 to −0.5), 1.3-log (95% CI −2.1 to −0.6), and 3.4-log (95% CI −4.7 to −2.2) CFU/mL after one, two, and three exposures, respectively (Figure 2d). Interestingly, ciprofloxacin at 0.4 μg/mL combined with ISP reduced biofilm-encased bacteria by a mean of 1.4-log (95% CI −2.1 to −0.6) CFU/mL after three exposures, indicating that this synergistic effect was dose-dependent. Thus, rifampicin antagonized while ciprofloxacin synergized with ISP in eliminating mature biofilm-encased MSSA.

### 3.2 Effect of phage ISP in combination with protein synthesis-targeting antibiotics on MSSA

#### 3.2.1 Planktonic phase MSSA

**Figure 3:**
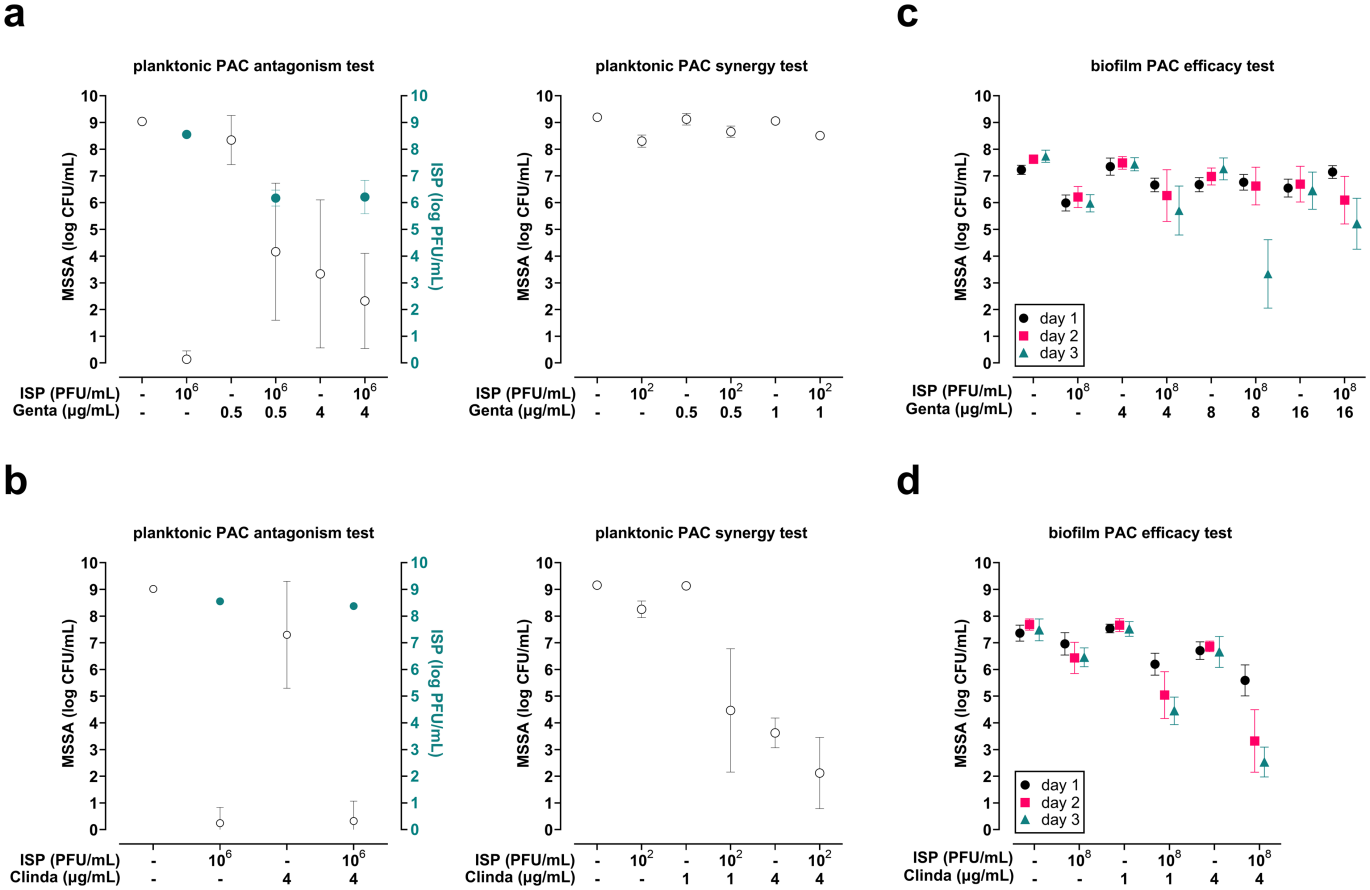
Efficacy of bacteriophage (phage) ISP, antibiotics targeting bacterial protein synthesis, and combinations thereof on planktonic and biofilm-encased methicillin-sensitive *Staphylococcus aureus* (MSSA). The efficacy of phage-antibiotic combinations (PAC) was tested by exposing MSSA in mid-logarithmic phase at 10^6^ CFU/mL to PAC with phage ISP at 10^6^ PFU/mL (multiplicity of infection (MOI) = 1) or ISP at 10^2^ PFU/mL (MOI = 0.0001) in combination with **(a)** gentamicin (Genta) or **(b)** clindamycin (Clinda) at suboptimal concentrations. After 24 h exposure (90 rpm, 37 °C), the bacteria were spun down by centrifugation (1,000 *xg*, 10 min). Thereafter, the phage titer (PFU/mL) in the supernatant was determined using the double agar overlay method, and the bacterial load in the pellet was assessed microbiologically after resuspension in 0.9% saline supplemented with 10 mM ammonium sulfate (II) hexahydrate (FAS; to neutralize residual phage activity). The open (white) symbols in **a** and **b** indicate the mean CFU/mL and the closed (blue) symbols indicate the mean PFU/mL. In addition, the efficacy of PAC was assessed on MSSA within seven-day mature biofilms on titanium-6% aluminum-7% niobium (Ti-6Al-7Nb) implant mimics. Biofilms were exposed daily to ISP at 10^8^ PFU/mL and its combination with **(c)** gentamicin or **(d)** clindamycin for up to three consecutive days. Before and after each exposure, planktonic bacteria were removed by two washes with 0.9% saline. Finally, MSSA were harvested from biofilms by sonication (40 kHz, 10 min) in 0.9% saline with 10 mM FAS for microbiological enumeration of the bacterial load. All exposures were diluted in brain-heart infusion broth. Results are from at least three independent experiments, each in triplicate. Data were log-transformed and error bars indicate the 95% confidence intervals.

Next, the effects of the protein-synthesis targeting antibiotics gentamicin and clindamycin were investigated in combination with ISP on planktonic phase MSSA (10^6^ CFU/mL). The results showed that gentamicin antagonized the effects of ISP (10^6^ PFU/mL, MOI = 1) on MSSA, and did not enhance the efficacy of ISP at suboptimal concentrations (10^2^ PFU/mL, MOI = 0.0001) (Figure 3a). Clindamycin at 4 μg/mL did not interfere with the killing capacity of ISP (10^6^ PFU/mL, MOI = 1). Moreover, this antibiotic at 1 μg/mL and 4 μg/mL in combination with ISP at suboptimal concentrations (10^2^ PFU/mL, MOI = 0.0001) synergistically and additively reduced the bacterial load by a mean 3.8-log (95% CI −5.8 to −1.8) and 1.5-log (95% CI −2.7 to −0.3) CFU/mL, respectively (Figure 3b). Thus, this data indicates synergistic antibacterial effects of the combination of clindamycin, but not gentamicin, with ISP against MSSA in planktonic phase.

#### 3.2.2 Biofilm-encased MSSA

The efficacy of repeated exposure to ISP (10^8^ PFU/mL) combined with gentamicin or clindamycin was then determined on biofilm-encased MSSA for up to three consecutive days. Repeated exposure to gentamicin at 4 and 16 μg/mL combined with ISP did not effectively reduce the bacterial load. However, gentamicin at 8 μg/mL combined with ISP synergistically reduced biofilm-encased bacteria after exposure for three consecutive days (Figure 3c). Further, clindamycin at 1 μg/mL combined with ISP reduced biofilm-encased MSSA by 0.8-log (95% CI −1.3 to −0.3), 1.4-log (95% CI −2.3 to −0.5), and 2.0-log (95% CI −2.5 to −1.5) CFU/mL after one, two, and three exposures, respectively (Figure 3d). This effect was even more pronounced when ISP was combined with clindamycin at 4 μg/mL, diminishing bacterial counts by 1.1-log (95% CI −1.7 to −0.5), 3.1-log (95% CI −4.2 to −2.0), and 3.9-log (95% CI −4.5 to −3.4) CFU/mL after one, two, and three exposures, respectively. This data shows that repeated exposures to ISP and clindamycin, but not gentamicin, effectively reduced biofilm-encased MSSA.

### 3.3 Effect of phage ISP combined with cell wall or cell membrane targeting antibiotics on MSSA

#### 3.3.1 Planktonic phase MSSA

**Figure 4:**
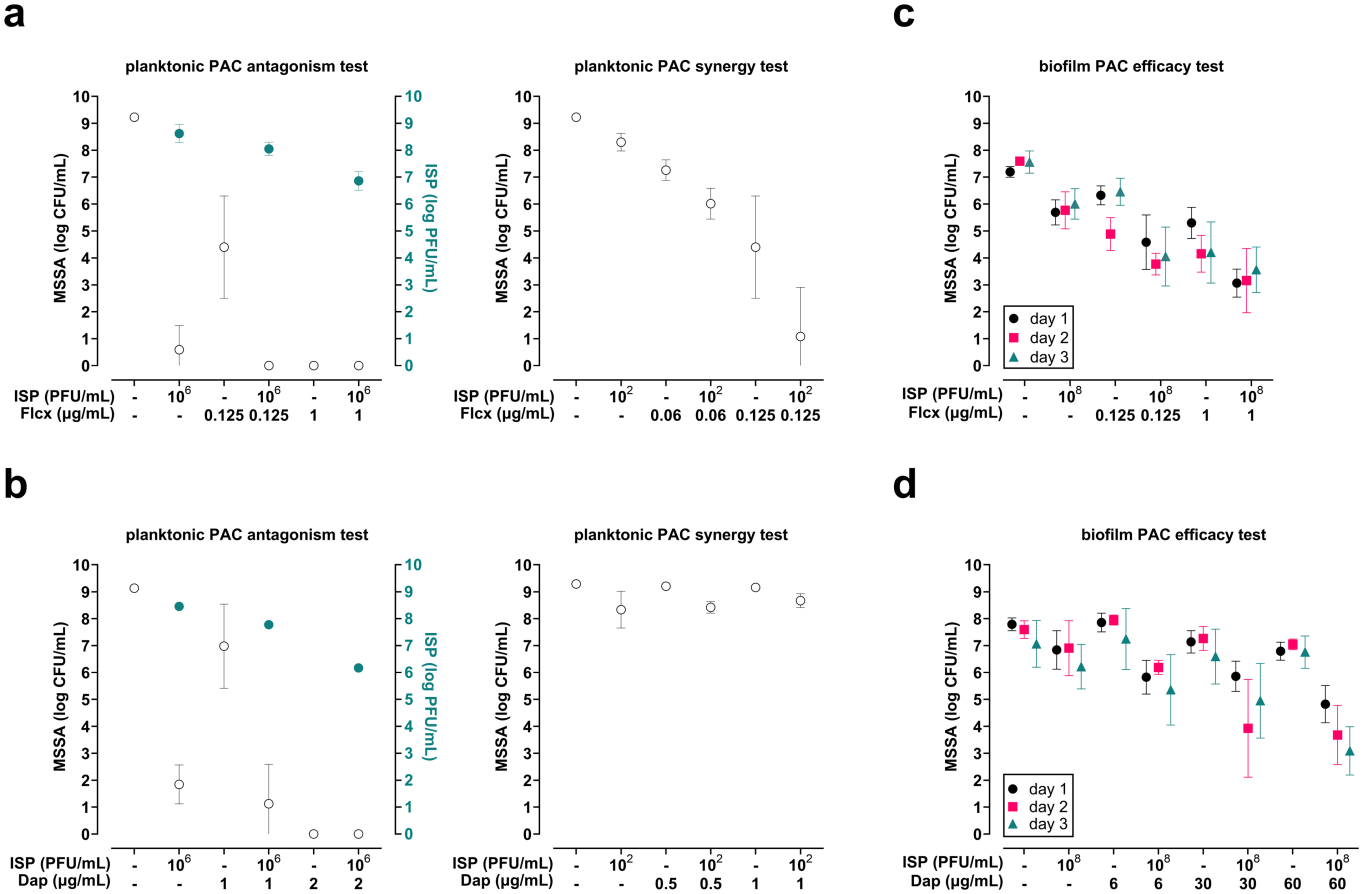
Efficacy of bacteriophage (phage) ISP, antibiotics targeting the bacterial cell wall or cell membrane, and combinations thereof on planktonic and biofilm-encased methicillin-sensitive *Staphylococcus aureus* (MSSA). The efficacy of phage-antibiotic combinations (PAC) was tested by exposing MSSA in mid-logarithmic phase at 10^6^ CFU/mL to PAC with phage ISP at 10^6^ PFU/mL (multiplicity of infection (MOI) = 1) or ISP at 10^2^ PFU/mL (MOI = 0.0001) in combination with **(a)** flucloxacillin (Flcx) or **(b)** daptomycin (Dap) at suboptimal concentrations. After 24 h exposure (90 rpm, 37 °C), the bacteria were spun down by centrifugation (1,000 x*g*, 10 min). Thereafter the phage titer (PFU/mL) in the supernatant was determined using the double agar overlay method, and the bacterial load in the pellet was assessed microbiologically after resuspension in 0.9% saline supplemented with 10 mM ammonium sulfate (II) hexahydrate (FAS; to neutralize residual phage activity). The open (white) symbols in **a** and **b** indicate the mean CFU/mL and the closed (blue) symbols indicate the mean PFU/mL. In addition, the efficacy of PAC was assessed on MSSA within seven-day mature biofilms on titanium-6% aluminum-7% niobium (Ti-6Al-7Nb) implant mimics. Biofilms were exposed daily to ISP at 10^8^ PFU/mL and its combination with **(c)** flucloxacillin or **(d)** daptomycin for up to three consecutive days. Before and after each exposure, planktonic bacteria were removed by two washes with 0.9% saline. Finally, MSSA were harvested from biofilms by sonication (40 kHz, 10 min) in 0.9% saline with 10 mM FAS for microbiological assessment of the bacterial counts. Brain-heart infusion broth was used as diluent for all exposures. Results are from at least three independent experiments, each in triplicate. Data were log-transformed and error bars indicate the 95% confidence intervals.

The effect of ISP combined with the cell wall-targeting antibiotic flucloxacillin and the cell membrane-targeting antibiotic daptomycin was tested on planktonic phase MSSA (10^6^ CFU/mL). The results showed that both flucloxacillin and daptomycin did not hamper the bactericidal activity of ISP (10^6^ PFU/mL, MOI = 1) against MSSA (Figures 4a,b). Flucloxacillin at 0.06 and 0.125 μg/mL, but not daptomycin, combined with ISP at suboptimal concentrations (10^2^ PFU/mL, MOI = 0.0001) effectively reduced bacterial counts by a mean 1.2-log (95% CI −1.8 to −0.7) and 3.3-log (95% CI −5.6 to −1.1) CFU/mL, respectively (Figure 4b). Accordingly, additive and synergistic effects were observed with the combination of flucloxacillin and ISP, whereas daptomycin and ISP showed indifferent effects.

#### 3.3.2 Biofilm-encased MSSA

Subsequently, biofilm-encased MSSA were daily exposed to ISP (10^8^ PFU/mL) in combination with flucloxacillin or daptomycin for up to three consecutive days. Flucloxacillin at 0.125 μg/mL combined with ISP reduced biofilm-encased bacteria by a mean 1.1-log (95% CI −2.1 to 0.1), 1.1-log (95% CI −1.8 to −0.5), and 2.0-log (95% CI −3.0 to −0.9) CFU/mL after one, two, and three exposures, respectively (Figure 4c). ISP combined with flucloxacillin at 1 μg/mL reduced the bacterial load by 2.2-log (95% CI −2.9 to −1.6), 1.0-log (95% CI −2.2 to −0.2), and 0.6-log (95% CI −1.5 to 0.2) CFU/mL after one, two, and three exposures, respectively (Figure 4c). One, two and three exposures of MSSA biofilms to ISP combined with daptomycin at 60 μg/mL reduced bacterial counts within biofilms by a mean 2.0-log (95% CI −2.7 to −1.3), 3.2-log (95% CI −4.5 to −1.9) and 3.1-log (95% CI −4.2 to −2.1) CFU/mL, respectively, while the combination of ISP and daptomycin at 6 μg/mL was indifferent against biofilm-encased MSSA (Figure 4d). Exposure to daptomycin at 30 μg/mL and ISP was similarly effective as ISP and daptomycin at 60 μg/mL for the first two consecutive days. However, the bacterial load increased by 1-log CFU/mL after three days exposure. Thus, repeated exposure of MSSA biofilms to ISP combined with flucloxacillin or high-dose daptomycin effectively reduced biofilm-encased bacterial counts.

### 3.4 Planktonic prediction of phage-antibiotic effects on biofilm-encased MSSA

**Figure 5:**
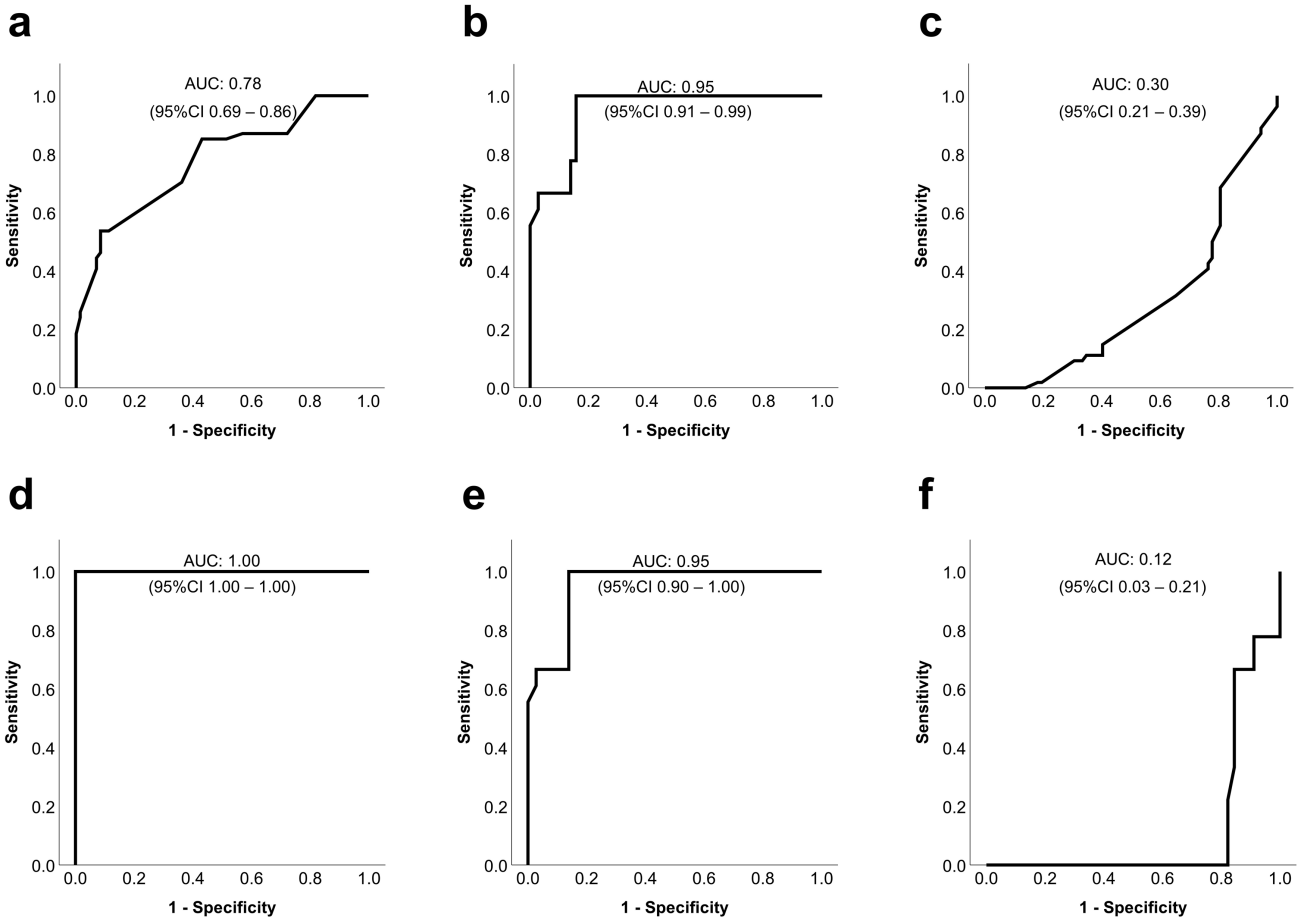
Performance of testing phage-antibiotic combinations (PAC) on planktonic methicillin-sensitive *Staphylococcus aureus* (MSSA) to predict the efficacy of PAC on biofilm-encased MSSA after three daily exposures. Receiver operating characteristic (ROC) curves were used to calculate whether the effects of PAC on planktonic phase MSSA were comparable to the effects of PAC on biofilm-embedded MSSA after daily exposures to the corresponding concentrations for three consecutive days. The graphs represent unfavorable (indifferent, antagonistic) effects **(a,d)**, antagonistic effects **(b,e)**, and synergistic effects **(c,f)** between antibiotics and phage ISP after three exposures on biofilm-encased MSSA predicted by planktonic bacteria. Graphs **a**, **b**, and **c** display the predictive capacity of all PAC concentrations tested, whereas **d**, **e,** and **f** are based on the values for PAC at low antibiotic concentrations that were tested on both planktonic and biofilm-encased MSSA. AUC = area under the curve. Data are from three experiments in triplicate, including two to three antibiotic concentrations per PAC in **a**, **b**, and **c**, and including one to two antibiotic concentrations per PAC in **d**, **e**, and **f**.

Lastly, ROC curves were calculated to determine whether testing the effects of PACs on planktonic phase MSSA correlate with their effects on biofilm-encased MSSA (Figure 1). The results revealed that the prediction of additive and synergistic effects was limited, reflected by AUC values of 0.32, 0.21, and 0.23 for one, two and three exposures, respectively. In contrast, predicting indifferent and antagonistic effects resulted in AUC values of 0.68, 0.79, and 0.78 for one, two, and three exposures, respectively (Figure 5a; Table 1). Specifically, results from planktonic testing predicted antagonistic effects accurately, reflected by AUC values of 0.89 after one and two exposures and 0.95 after three exposures, sensitivity values of 0.73, 0.78, 1.00, and specificity values of 0.95, 0.90, and 0.82 for one, two, and three exposures, respectively (Figure 5b; Table 2). Synergistic effects on biofilm-encased bacteria, on the other hand, were poorly predicted by planktonic testing of PAC, indicated by AUC values of 0.41, 0.55, and 0.30 for one, two, and three exposures, respectively (Figure 5c; Table 2).

**Table 1:** Prediction of additive and synergistic effects, or indifferent and antagonistic effects of phage-antibiotic combinations (PAC) on biofilm-encased methicillin-sensitive *Staphylococcus aureus* (MSSA) using planktonic MSSA.

Daily biofilm exposure	Day 1		Day 2		Day 3	
	All	Low	All	Low	All	Low
AUC additive and synergistic	0.32	0.49	0.21	0.12	0.23	0.00
95% CI	0.22–0.41	0.34–0.64	0.13–0.29	0.03–0.21	0.14–0.31	0.00
AUC indifferent and antagonistic	0.68	0.51	0.79	0.88	0.78	1.00
95% CI	0.59–0.78	0.37–0.66	0.71–0.87	0.79–0.97	0.69–0.86	1.00–1.00
Sensitivity	0.46	N/A	0.6	1.00	0.53	0.94
Specificity	0.73	0.62	0.87	1.00	0.85	1.00

Additive and synergistic effects represent favorable effects of PAC, while indifferent and antagonistic effects indicate unhelpful effects of PAC. The area under the curve (AUC), sensitivity, and specificity were determined using the datasets for phages combined with the values for all tested antibiotic concentrations (“all”) and the datasets for phage combined with low antibiotic concentrations tested on both planktonic and biofilm-encased MSSA (“low”). Data are from three experiments in triplicate, with “all” including two to three antibiotic concentrations and “low” including one to two antibiotic concentrations per PAC. N/A = not applicable, due to absence of values with an effect.

**Table 2:** Prediction of antagonistic or synergistic effects of phage-antibiotic combinations (PAC) on biofilm-encased methicillin-sensitive *Staphylococcus aureus* (MSSA) using planktonic MSSA.

Daily biofilm exposure	Day 1		Day 2		Day 3	
	All	Low	All	Low	All	Low
AUC antagonistic effects	0.89	0.95	0.89	0.95	0.95	0.95
95% CI	0.84–0.95	0.90–1.00	0.82–0.95	0.90–1.00	0.91–0.99	0.90–1.00
Sensitivity	0.73	0.85	0.78	0.85	1.00	1.00
Specificity	0.95	1.00	0.90	1.00	0.82	0.86
AUC synergistic effects	0.41	N/A	0.55	N/A	0.30	0.12
95% CI	0.29–0.53	N/A	0.45–0.64	N/A	0.21–0.39	0.03–0.21
Sensitivity	0.39	N/A	0.11	N/A	0.39	0.44
Specificity	0.74	N/A	0.68	N/A	0.81	0.84

The area under the curve (AUC), sensitivity and specificity were determined using the datasets for phages combined with the values for all tested antibiotic concentrations (“all”) and the datasets for phages combined with low antibiotic concentrations tested on both planktonic and biofilm-encased MSSA (“low”). Data are from three experiments in triplicate, with “all” including two to three antibiotic concentrations and “low” including one to two antibiotic concentrations per PAC. N/A = not applicable, due to absence of values with an effect.

Next, to improve the ability of planktonic PAC testing to predict the effects on biofilm-encased MSSA, data for PAC at high antibiotic concentrations that could not be tested on planktonic phase bacteria were excluded from the prediction analysis. Although this approach did not improve the prediction of additive and synergistic effects, the results showed that the AUC values to predict antagonistic and indifferent effects increased to 0.88 and 1.00 for two and three exposures, respectively. In addition, the sensitivity and specificity both increased to 1.00 after two exposures, and to 0.94 and 1.00 after three exposures (Figure 5d; Table 1). Excluding the high antibiotic values increased the sensitivity and specificity for predicting antagonistic effects to 0.85 and 1.00 (one and two exposures), and to 1.00 and 0.86 (three exposures). This increase was not observed for synergy, as reflected by AUC values of 0.12 after three exposures (Figures 5e,f; Table 2). In summary, PAC testing on planktonic MSSA correctly predicted antagonistic, but not synergistic effects on biofilm-encased bacteria for the respective PAC concentrations.

## 4 Discussion

The frequent failure of single antibiotic treatment to eliminate biofilm-associated infections has led to combining agents with different modes of action, such as phages and antibiotics, alongside surgical debridement of the infected and often necrotic tissue. As screening PAC on mature bacterial biofilms is time-consuming, this study investigated whether the effects of phage ISP combined with antibiotics on biofilm-encased MSSA could be predicted by testing their effects on the planktonic counterparts. The interactions of PACs were consistent across both systems: two of six antibiotics antagonized the actions of ISP, one antibiotic showed no interactions with the phage, and three of six antibiotics synergized with ISP. In conclusion, PAC testing on planktonic MSSA accurately predicted antagonistic effects, but not synergistic effects for the respective concentrations of PACs on biofilm-encased MSSA.

Several findings of our study support the latter. Our microbiological assays indicated a similar trend in effects for the respective PACs against planktonic and biofilm-encased MSSA. Although planktonic PAC testing accurately predicted antagonistic effects on biofilm-encased bacteria, this approach could not predict synergistic anti-biofilm effects. Accordingly, PAC at high daptomycin and gentamicin (only 8 μg/mL) concentrations effectively reduced biofilm-encased bacteria. At the same time, these effects were not predicted by the suboptimal antibiotic concentrations tested on planktonic phase bacteria. Further, higher concentrations of almost all tested antibiotics were needed to elicit an effect on biofilm-encased bacteria compared to their planktonic counterparts. This difference in antibiotic and phage sensitivity illustrates the challenge to translate planktonic effects to biofilms at high antibiotic concentrations. Biofilm-encased bacteria exhibit considerable tolerance to phages and antibiotics, which means that they require exposure to higher concentrations than planktonic bacteria to reduce the bacterial burden (Ceri et al., 1999).

Repeated exposure to ISP combined with ciprofloxacin, clindamycin, flucloxacillin or high-dose daptomycin synergistically reduced biofilm-encased MSSA compared to a single exposure. Moreover, predicting the effects of PAC on MSSA in biofilms using planktonic bacteria was most accurate when the results from two to three consecutive PAC exposures on biofilms were considered. Many antibiotics become immobilized within biofilms by binding to the matrix components, limiting the free antibiotic concentration within the biofilm (Liu et al., 2024). Repeated dosing of PAC may saturate antibiotic binding to these matrix components, thereby increasing free antibiotic concentrations within the biofilm and enhancing antibiotic biofilm penetration. Possibly, repeated dosing increases the local, unbound PAC concentration to levels initially tested on planktonic bacteria, resulting in the enhanced specificity of our prediction model. This would also explain the observation that exposure to flucloxacillin alone, characterized by its high protein binding (Røder et al., 1995), further reduced biofilm-encased bacteria after two exposures by increasing the pharmacologically active fraction of the antibiotic.

In line with our findings, a previous study showed that the PACs utilizing phage Sb-1 were less effective in reducing biofilm-embedded bacteria than the planktonic counterparts (Kebriaei et al., 2022). To our knowledge, we have been the first to directly link PAC efficacy on planktonic bacteria to biofilm-encased bacteria using a predictive model. Interestingly, Shaban *et al.* developed a machine-learning based approach to predict the *in vitro* effects of various antibiotics on biofilms (Shaban and Alkawareek, 2022). The development of machine-learning models for PACs can potentially enhance the efficacy predictions of PACs on biofilms using planktonic bacteria.

Considering the nature of interactions between phages and antibiotics, previous research indicated that these interactions largely depend on the antibiotic’s mechanism of bacterial inhibition, the concentrations used, the phage, and the host microenvironment (Gu Liu et al., 2020; Kim et al., 2024; Akturk et al., 2019). Hence, this study combined ISP with antibiotics targeting different cellular processes or structures: bacterial transcription, translation, cell wall, and cell membrane. In line with previous observations, our results demonstrate that phage-antibiotic interactions depend on the antibiotic class the phage is combined with (Kim et al., 2024).

As to the mechanisms underlying the synergistic and additive phage-antibiotic interactions, phages may enhance the antibiotic susceptibility of bacteria (Gu Liu et al., 2020). For example, beta-lactam antibiotics (e.g., flucloxacillin) and fluoroquinolones can induce bacterial cell filamentation, thereby increasing the bacterial cell surface area and their susceptibility to phages (Bulssico et al., 2023). In agreement, clinically suboptimal concentrations of ciprofloxacin, clindamycin, and flucloxacillin in the presence of ISP were more effective against MSSA than in its absence. Others suggested that suboptimal antibiotic concentrations can enlarge the plaque size and delay lysis, resulting in increased phage production and synergistic reductions in bacterial counts (Morrisette et al., 2020). Since the phage production by synergistic PAC was not increased compared to the phage-only control, the enhanced antibacterial effect is more likely to be attributed to the improved antibiotic efficacy in the presence of ISP than enhanced phage production.

The concept of phage-antibiotic synergy is based on the evolutionary principle that combining two approaches that induce different selective pressures is often more effective than a single approach (Torres-Barceló and Hochberg, 2016). Conversely, phage-antibiotic antagonism may occur when the two approaches target similar mechanisms. This principle explains the observed antagonistic interaction between rifampicin and ISP, as rifampicin hampers the transcription of ISP by targeting the bacterial RNA polymerase. Notably, a previous study showed that phage Sb-1, which encodes RNA polymerase, synergized with rifampicin against *S. aureus* biofilms (Tkhilaishvili et al., 2018). Given that rifampicin is frequently used to treat *S. aureus* implant-associated infections (Espíndola et al., 2022; Zimmerli and Sendi, 2019), these findings underscore the importance of testing PACs with antibiotics such as rifampicin.

Additionally, antibiotics that target protein translation may reduce phage production by hampering their amplification (Pons et al., 2023). Gentamicin, for example, binds to the 16S rRNA of the 30S ribosomal subunit, which may result in mistranslation and the generation of non-functional phage proteins (Zuo et al., 2021). Indeed, gentamicin hindered the amplification of ISP and its efficacy on planktonic phase bacteria, and repetitive exposures to ISP and gentamicin at 4 and 16 μg/mL did not further reduce biofilm-encased *S. aureus*. In contrast, other studies have reported synergistic reductions in *S. aureus* biofilms upon repeated exposure to phages and gentamicin (Mannala et al., 2024; Akturk et al., 2023). The phages, bacterial strains, dosages, approaches, and models differ from those in our study, and these differences can greatly influence the treatment outcomes. For instance, Akturk *et al.* utilized a dual species biofilm in an *in vitro* wound model and different dosing intervals (Akturk et al., 2023), while Mannala *et al.* utilized 24 h biofilms on K-wire in *Galleria mellonella* (which also have an innate immune system (Wojda, 2017)), sequential treatment, and high phage MOI (Mannala et al., 2024). Unexpectedly, we observed here that ISP and gentamicin at 8 μg/mL significantly reduced the bacterial load within biofilms after three days of exposure. There might be an optimal gentamicin concentration that can overcome the antagonistic interaction through repetitive dosing, underscoring the need to test a range of phage-antibiotic concentrations. Investigating the interaction between phages and gentamicin is especially relevant for metal implants fixed with bone cement, which commonly incorporates gentamicin (Cara et al., 2020).

Interestingly, the protein translation-targeting antibiotic clindamycin did not interfere with the translation of ISP. Rather, clindamycin synergized with ISP against planktonic phase *S. aureus*, in line with previous findings (Van Nieuwenhuyse et al., 2021), and against biofilm-encased bacteria. This efficacy indicates that the reduced ribosome assembly, caused by clindamycin’s binding to the 23S rRNA of the 50S ribosomal subunit, is still sufficient for amplification of ISP. This phenomenon has also been observed for other phages combined with clindamycin at sub-inhibitory concentrations against *S. aureus* biofilms (Liu et al., 2021).

This study has several limitations. The immune system and microenvironment of the host were not incorporated, while these factors influence the efficacy of PAC (Gu Liu et al., 2020). For instance, plasma and synovial fluid can reduce the phage activity (Mutti et al., 2023), and host-mimicking conditions can reduce bacterial growth rates and thereby impair the effectiveness of PACs (Gu Liu et al., 2020). While the absence of these factors in our model may lead to an overestimation of the treatment efficacy of PACs, it is important to note that PACs that show poor *in vitro* effects in the absence of host-mimicking factors are unlikely to be effective in their presence. The planktonic PAC testing conducted in this study primarily serves to exclude PACs that antagonize, and subsequently to select favorable PACs that, if needed, can be further assessed under more representative conditions. Previous studies have demonstrated complexity and presence of tolerant subpopulations in biofilms cultured for seven days *in vitro* (Verheul et al., 2024; van Dun et al., 2023). Nevertheless, *in vivo* biofilms are not fully represented by *in vitro* biofilm models that lack host-mimicking factors such as shear stress, physiological fluids, and immune cells.

This study utilized a single phage and a single bacterial strain, serving as a proof of concept. However, generalization of PAC effects is limited by the genetic and phenotypic diversity of both phages and bacteria. This indicates that the interactions need to be characterized for each PAC and bacterial strain. Moreover, a better understanding of the precise interactions between phages, antibiotics, and their combinations with bacteria may lead to more reliable predictions of PAC effects on specific bacterial strains. Further, previous studies showed that PAC exposure prevented the emergence of phage-resistant bacteria (Gu Liu et al., 2020; Kebriaei et al., 2022). Our CFU counts did not suggest the proliferation of resistant mutants, however we did not assess the emergence of resistance in the surviving biofilm fraction after repeated PAC exposure.

Additionally, the precise antibiotic and phage concentrations to which the biofilm-encased bacteria were exposed remain unclear, as it is unknown which concentrations of these agents penetrate the biofilm due to its protective matrix (Verheul et al., 2024). Although phage suspensions containing 10^8^–10^10^ PFU/mL have been administered to patients (Aslam et al., 2020), the bacterial load *in vivo* is often unknown as well as the number of phages encountered by the biofilm-embedded bacteria. It should be noted that the antibiotic concentrations used in this study are clinically reachable in the circulation. However, the concentrations of these agents at the infection sites often remain elusive. Importantly, the ability of these agents to reach the site of infection may be hampered by impaired vascularity in patients with bone and joint infections. Understanding the concentrations that can be reached at the site of infection will enhance the predictive value of phage-antibiotic effects on bacteria.

Hence, future studies are needed to elucidate the pharmacokinetics and pharmacodynamics of PAC for these infections. Furthermore, the range of concentrations of the antimicrobial agents could be extended by using less labor-intensive readouts than CFU assays, i.e., checkerboards. Finally, further studies should prioritize testing the efficacy of PAC in advanced *in vitro* models, validate the effects clinically, and refine strategies through multidisciplinary collaborations.

Combination therapies like PAC can enhance antibiotic efficiency when addressing biofilm-associated infections, reduce resistance development, and may improve patient outcomes (Kunz Coyne et al., 2024; Morrisette et al., 2020; Fedorov et al., 2023). Here, PAC testing on planktonic bacteria was particularly useful for predicting antagonistic effects, which aligns with primum non nocere (i.e., first, do no harm) in the clinic. In other words, the rapid and easy testing of PAC on planktonic bacteria may significantly reduce the risk of adverse effect in patients, while underestimating additive and synergistic effects upon repeated exposure to higher PAC concentrations. Together, planktonic PAC testing provides an important first-line screening prior to starting PAC treatment in patients with biofilm-associated infections.

## Data Availability

The raw data supporting the conclusions of this article will be made available by the authors, without undue reservation.
